# The burden of acute eye conditions on different healthcare providers: a retrospective population-based study

**DOI:** 10.3399/BJGP.2022.0616

**Published:** 2024-03-05

**Authors:** Anna Rawlings, Angharad E Hobby, Barbara Ryan, Andrew Carson-Stevens, Rachel North, Mathew Smith, Sioned Gwyn, Nik Sheen, Jennifer H Acton

**Affiliations:** Swansea University Medical School, Swansea University, Swansea.; School of Optometry and Vision Sciences, Cardiff University, Cardiff, and University of the West of England, Bristol;; School of Optometry and Vision Sciences, Cardiff University, Cardiff.; PRIME Centre Wales, Division of Population Medicine, School of Medicine, Cardiff University, Cardiff.; School of Optometry and Vision Sciences and PRIME Centre Wales, Division of Population Medicine, School of Medicine, Cardiff University, Cardiff.; School of Pharmacy and Pharmaceutical Sciences, Cardiff University, Cardiff.; PRIME Centre Wales, Division of Population Medicine, School of Medicine, Cardiff University, Cardiff.; Health and Education Improvement Wales (HEIW), Nantgarw.; School of Optometry and Vision Sciences, Cardiff University, Cardiff.

**Keywords:** emergency medicine, eye, general practice, optometry, pharmacy, primary health care

## Abstract

**Background:**

The demand for acute eyecare exponentially outstrips capacity. The public lacks awareness of community eyecare services.

**Aim:**

To quantify the burden of acute eyecare on different healthcare service providers in a national population through prescribing and medicines provision by GPs, optometrists, and pharmacists, and provision of care by accident and emergency (A&E) services. A secondary aim was to characterise some of the drivers of this burden.

**Design and setting:**

A retrospective data-linkage study set in Wales, UK.

**Method:**

Analysis of datasets was undertaken from the Secure Anonymised Information Linkage Databank (GP and A&E), the Eye Health Examination Wales service (optometry), and the Common Ailments Scheme (pharmacy) during 2017–2018.

**Results:**

A total of 173 999 acute eyecare episodes delivered by GPs (168 877 episodes) and A&E services (5122) were identified during the study. This resulted in 65.4 episodes of care per 1000 people per year. GPs prescribed a total of 87 973 653 prescriptions within the general population. Of these, 820 693 were related to acute eyecare, resulting in a prescribing rate of 0.9%. A total of 5122 eye-related and 905 224 general A&E attendances were identified, respectively, resulting in an A&E attendance rate of 0.6%. Optometrists and pharmacists managed 51.8% (116 868) and 0.6% (2635) of all episodes, respectively. Older females and infants of both sexes were more likely to use GP prescribing services, while adolescent and middle-aged males were more likely to visit A&E. GP prescribing burden was driven partially by economic deprivation, access to services, and health score. Season, day of the week, and time of day were predictors of burden in GP and A&E.

**Conclusion:**

Acute eyecare continues to place considerable burden on GP and A&E services in Wales, particularly in urban areas with greater economic deprivation and lower overall health. This is likely to increase with a rapidly ageing population. With ongoing pathway development to better utilise optometry and pharmacy, and improved public awareness, there may be scope to change this trajectory.

## Introduction

The demand for urgent eyecare exceeds capacity,^1^^,^^2^ but continues to increase.^3^ Eye-related attendances to UK accident and emergency (A&E) departments rose from 1.9% in 2014 to 3.8% in 2015,^4^ and a nationally representative sample in the US rose from 740 000 in 2007 to 932 000 in 2015.^5^ More recently, the burden on eyecare services has been further complicated by the COVID-19 pandemic.^6^^,^^7^

Eye-related issues account for 1%–2% of all GP consultations.^8^^,^^9^ Conjunctivitis is the most common ocular disorder presenting to GPs.^10^^,^^11^ Yet, many GPs are not confident in the management of red eye conditions.^12^

Public awareness as to where to seek help for eye conditions is lacking.^13^^,^^14^ While many ocular problems are acute, a high proportion are not sight- or life-threatening. Non-emergency conditions comprised 44% of all eye-related US emergency department visits.^15^ In the UK, 30% and 37% attending specialist ocular^16^ and general A&E,^17^ respectively, could have been managed in the community. Effective optometrist-led enhanced eyecare^4^^,^^18^^–^^21^ and pharmacist-led minor conditions schemes^22^^,^^23^ could reduce the burden on hospital and GP services.^24^

**Table:** How this fits in

The demand for acute eyecare is high, and new eyecare pathways delivered by optometrists and pharmacists are promising, yet the burden of acute eyecare for all acute eyecare providers is unknown. This is the first study to collate data at a national level and describe attendance and prescribing rates in Wales. Optometrists and GPs managed the greatest burden, but significant A&E attendances remain. Improving public awareness of the allied healthcare services for acute eye conditions may help to relieve current pressures.

**Figure 1.**
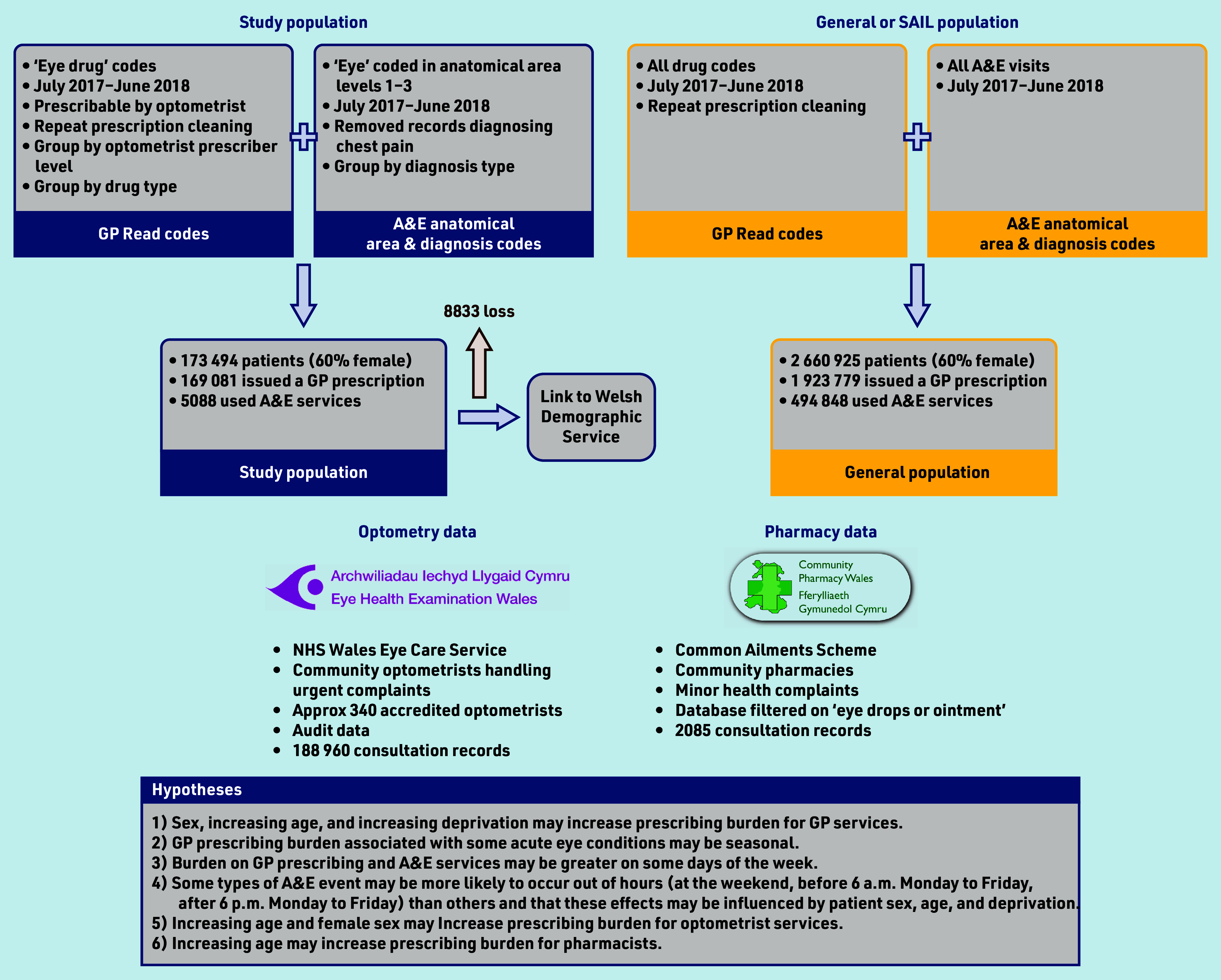
Study flowchart and hypotheses. A&E = accident and emergency. SAIL = Secure Anonymised Information Linkage.

The study primarily aimed to quantify the burden of acute eyecare on different healthcare service providers in a national population, including the quantification of burden on GP, optometry, and pharmacy prescribing and medicines provision, and on A&E services. A secondary aim was to understand some of the drivers of increasing burden. Several hypotheses were established, which are illustrated in Figure 1.

## Method

### Study design and data sources

This was a cross-sectional study of patients presenting to GP, A&E, community pharmacy, or optometry services in Wales for acute eyecare. Anonymised patient and consultation data, demographic data, and socioeconomic data were acquired from the Secure Anonymised Information Linkage (SAIL) Databank,^25^^,^^26^ the Eye Health Examination Wales service, and the Common Ailments Scheme. The study flowchart is presented in Figure 1, showing data sources used in this study (Supplementary Information gives detailed description of data sources).

The study took the 2 660 925 people registered to a SAIL GP practice at any point between 1 July 2017 and 30 June 2018 (study period), equating to 85.1% of the mid-2017 whole-Wales population.^27^ The following two sub-sets of this population were considered: the study population comprised all individuals who had interacted with a SAIL GP or Welsh A&E unit for acute eyecare during the study period; and the general population comprised all individuals who were issued a prescription by a SAIL GP or attended a Welsh A&E unit for any reason, including for acute eyecare, during the study period.

Licensed medicines for acute eye conditions can be prescribed by a GP, independent prescriber (IP) optometrist, or another prescriber, and a sub-set of these can be supplied by UK entry-level optometrists. GP and A&E datasets were selected based on coded medicines or items associated with acute eyecare (Supplementary Information provides a description of the coding processes). The final code lists were deposited in the SAIL Databank Concept Library^28^ (phenotypes PH1237 and PH1238).^29^

The Welsh Government’s measurement of deprivation is the Welsh Index of Multiple Deprivation (WIMD; Supplementary Information provides a detailed description of WIMD). The following deprivation information was captured for each individual based on their residential address on the date of their first eligible event in the study: overall deprivation score; access to services (ATS) domain score in quintiles (ATS levels 1–5, level 1 being the most deprived); and health domain score. Publicly available metrics for the ATS and health domains for each individual consisted of the following: 1) average public and private travel times to GPs and pharmacies for each residential area (minutes); and 2) the rate of chronic and limiting conditions^14^ for each residential area, respectively. Supplementary Information provides the calculation of age.

### Incidence and prescribing rates

Rate of incidence of acute eye episodes per 1000 persons registered to SAIL GPs were calculated within the study period. The rate of eligible GP-issued prescriptions was calculated from the total number of prescriptions issued for all conditions by SAIL GPs during the same period.

### Statistical analysis

Data visualisation was used to understand proportional changes in burden over temporal, demographic, and socioeconomic ranges. Statistical models were used to investigate whether these changes were statistically significant and to understand some of the drivers underpinning them. The study has reported on models of best fit, as assessed by likelihood ratio tests (lmtest),^30^ and with the greatest predictive power assessed by comparison of McFadden’s pseudo-*R*^2^ (DescTools).^31^

Hierarchical quasi-Poisson generalised linear model (GLM) modelled effects of demographics (age and sex) and socioeconomic status (WIMD quintiles) on the total number of GP prescribing events per person (total burden). Patient ages were grouped only for the purposes of data visualisation and to protect privacy of patient information.

Hierarchical binomial and multinomial logistic regression (nnet Package)^32^ modelled the timings of first prescription issued by GPs and of the first A&E event attended. Temporal predictors were time of day, day of week, and season in which these first events took place. For each temporal predictor examined in each service provider, likelihood of an event occurring was considered at the following two levels: total burden wherein all event types were combined (binomial logistic regressions); and individual drug or diagnosis type (multinomial logistic regressions).

Data linkage and cleaning was performed using Structured Query Language (SQL) to query IBM Db2 databases, and analysis and data visualisation using R (version 4.1.2).

## Results

### Demographics of primary and secondary acute eyecare provision in Wales

The general population with any GP event was 1 923 779 (50.2% female), while the total study population receiving acute eyecare via GP and A&E services in that year was 173 494 (60.4% female; Table 1).

**Table 1.** Overview of the general and study populations selected for the study broken down by sex and Welsh Index of Multiple Deprivation (WIMD) score

	**General population**	**General population with any GP event**	**General population with any A&E event**	**Study population**	**Study population with eye-related GP event**	**Study population with eye-related A&E event**
**Total count**	**2 660 925**	**1 923 780**	**494 848**	**173 494**	**169 081**	**5088**

**Sex**						
Male	1 325 255	867 310	246 718	68 737	65 776	3320
Female	1 335 664	1 056 469	248 130	104 757	103 305	1768
Missing	6	1	0	0	0	0

**Deprivation score**						
WIMD 1 (most deprived)	506 137	385 821	115 245	34 649	33 557	1268
WIMD 2	491 846	376 240	103 187	33 632	32 764	1007
WIMD 3	453 482	338 633	85 419	31 449	30 757	784
WIMD 4	429 544	323 647	75 709	29 980	29 270	802
WIMD 5 (least deprived)	485 449	365 009	78 375	34 951	34 134	965
Missing	294 467	134 430	36 913	8833	8599	262

*General population = all individuals registered to a SAIL GP practice at any time during the study period (85.1% of the mid-2017 whole Wales population); study population = individuals within the general population who had an eligible GP prescribing event or A&E event during the study period. A&E = accident and emergency. SAIL = Secure Anonymised Information Linkage.*

**Figure 2.**
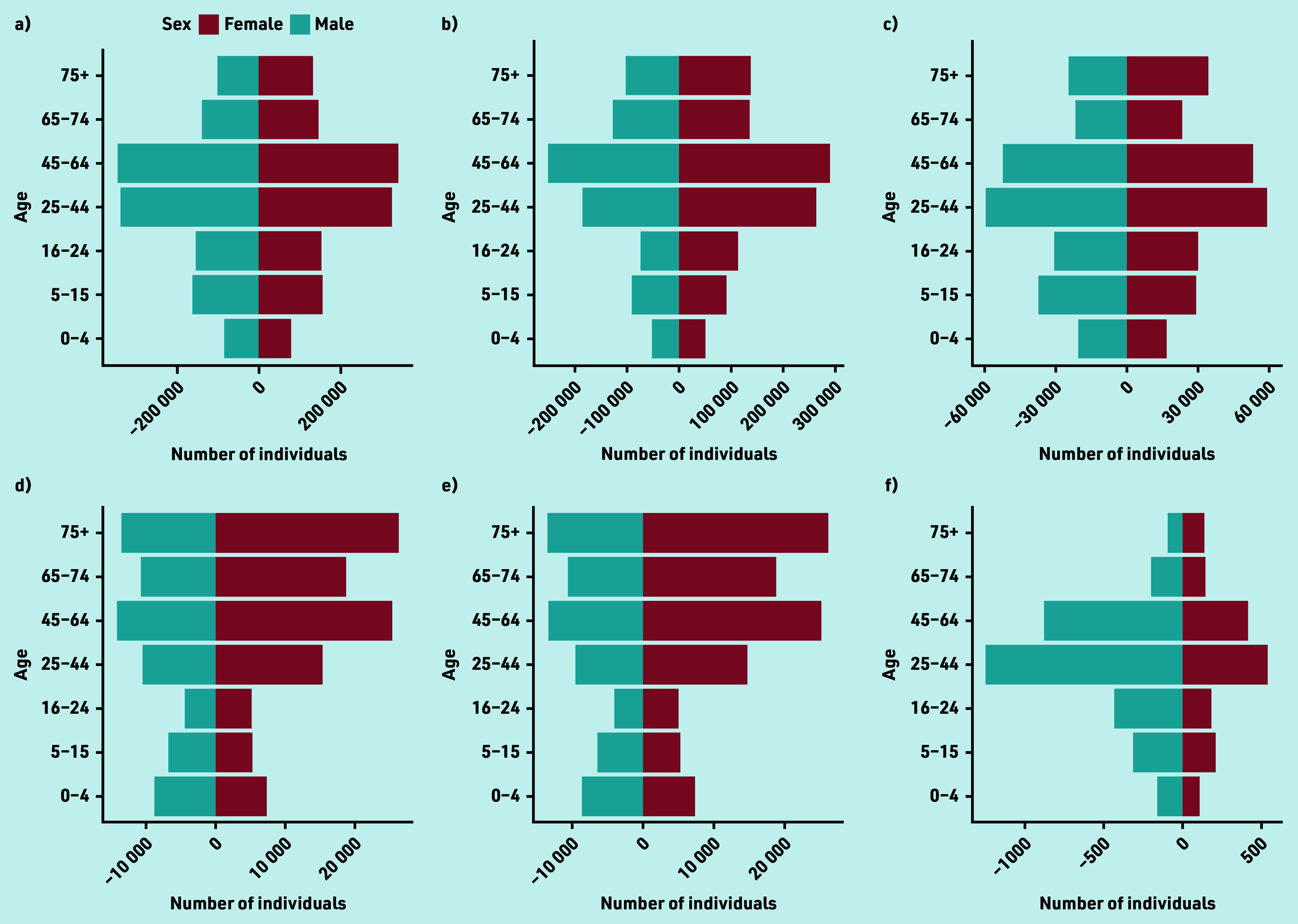

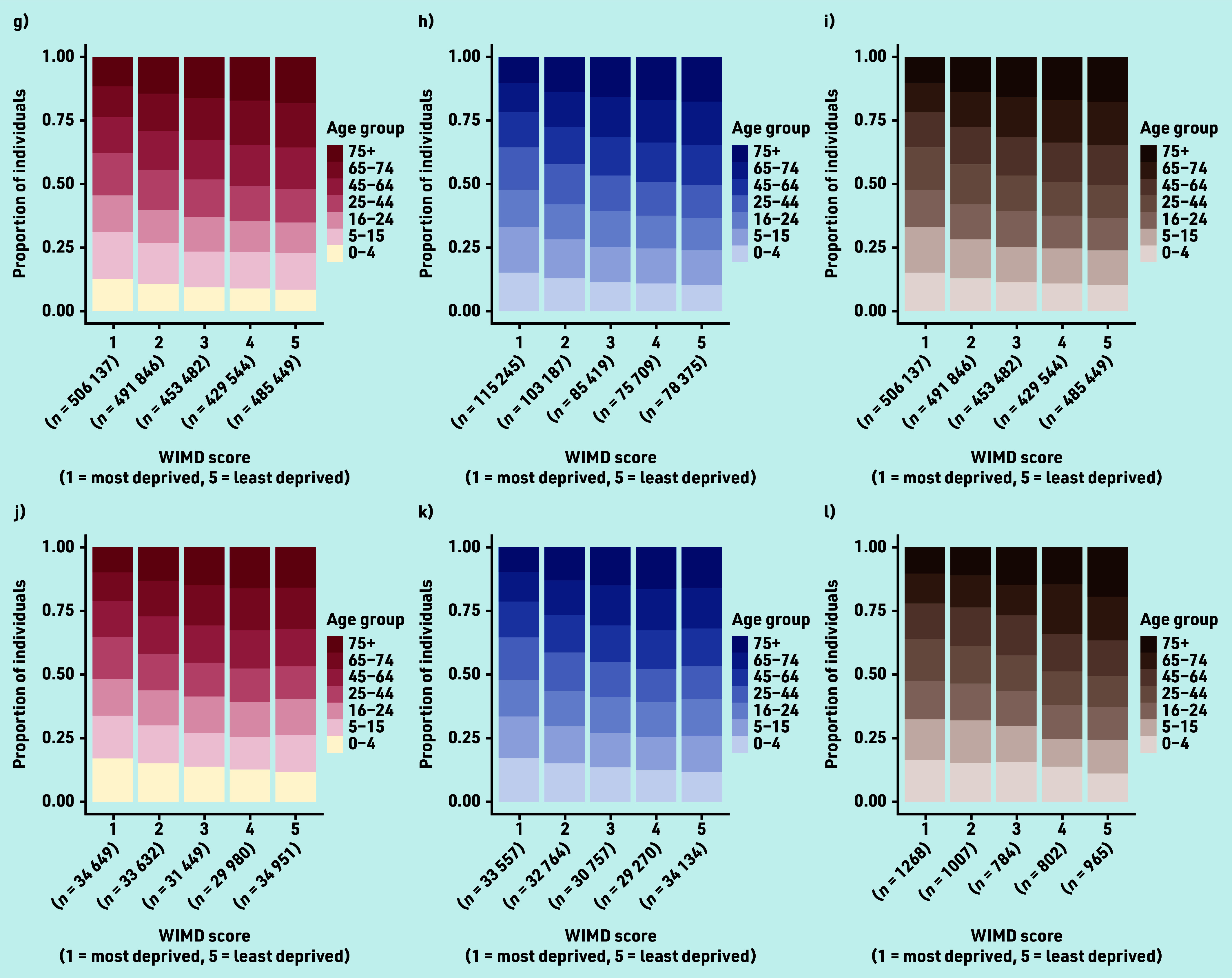
**(a–f).** GP and A&E dataset showing distribution with age (a–f). The number of male and female individuals in each of seven age groups are shown within (a) the general Wales population, (b) the general Wales population who had a GP prescribing event for any condition during the study period, (c) the general Wales population who used A&E services for any reason during the study period, (d) the study population, (e) the study population who had a GP prescribing event for an acute eye condition during the study period, and (f) the study population who used A&E services for an eye-related injury during the study period. The age groupings are based on those used by the Welsh Government in the derivation of population estimates (https://statswales.gov.wales). A&E = accident and emergency. WIMD = Welsh Index of Multiple Deprivation. **(g–l).** GP and A&E dataset showing distribution with Welsh Index of Multiple Deprivation (g–l). The proportion of individuals within each of seven age groups within WIMD quintiles are shown in (g) the general Wales population, (h) the general Wales population who had a GP prescribing event for any condition during the study period, (i) the general Wales population who used A&E services for any reason during the study period, (j) the study population, (k) the study population who had a GP prescribing event for an acute eye condition during the study period, and (l) the study population who used A&E services for an eye-related injury during the study period. The age groupings are based on those used by the Welsh Government in the derivation of population estimates (https://statswales.gov.wales). A&E = accident and emergency. WIMD = Welsh Index of Multiple Deprivation.

The study population using GP services for eyecare (Figure 2e) was dominated by individuals aged >25 years. Females used these services more than males in all age groups. A&E services (Figure 2f) were used most by those aged 25–64 years and all age groups up to 74 years were dominated by males.

Burden of eyecare on GP and A&E services decreased in younger individuals (0–44 years) as deprivation reduced and there was an increase in burden in the oldest age groups (65+) with reduced deprivation, mirroring trends for conditions generally (Figure 2g–l).

### Incidence and prescribing rates

A total of 173 999 acute eyecare episodes delivered by GPs (168 877 episodes) (episode numbers not tabulated) and A&E services (5122) were identified during the study period. This resulted in an incidence rate of 65.4 episodes of care per 1000 people per year. There were 300 referrals between GP and A&E services. Supplementary Information provides detailed results on referrals between services.

GPs prescribed a total of 87 973 653 prescriptions within the general population. Of these, 820 693 were related to acute eyecare (Table 2), resulting in a prescribing rate of 0.9% for acute eye conditions for the year. Most eye-related prescriptions were for ocular lubricants (623 250) or anti-allergy or anti-inflammatory drugs (114 766). A total of 766 083 (93.3%) could be supplied by entry-level optometrists while 54 610 (6.7%) prescriptions would require IP qualification. Overall, mean GP episode burden was 3.65 prescribing events per person and 63.1% of episodes comprised a single prescription.

**Table 2.** (a) GP prescribing and (b) A&E events in the study and general population

**a)**	**GP eyecare patients**	**GP eyecare prescriptions**	**Maximum prescriptions per patient year**	**Mean prescriptions per patient per year**

**Total count**	**185 716**	**820 693**	**28**	**3.65**

**Prescription type**				
Anti-allergy and anti-inflammatory drugs	41 347	114 766	17	2.55
Antimicrobial drugs	62 462	80 921	14	1.19
Ocular lubricants	81 339	623 250	26	5.60
Physical treatments	422	1609	14	3.78
Other	146	147	1	1.01

**Equivalent prescriber level**				
Entry level	166 371	766 083	26	3.78
Independent prescriber	22 480	54 610	17	2.30

**b)**	**A&E eyecare patients**	**A&E eyecare events**		

**Total count**	**5088**	**5122**		

**Diagnosis**				
Allergy	17	17		
Foreign body	1207	1241		
Ophthalmic	940	946		
Other — non-serious	2339	2377		
Other — serious	635	641		

**Treatment**				
Bandage or support	16	16		
Dressing	75	75		
Drug administration	564	566		
Guidance or advice only	1312	1341		
Incision and drainage	7	7		
No treatment required	5	5		
Not stated	2655	2685		
Observation	14	14		
Removal of foreign body	370	376		
Wound closure	136	137		

*Total patient count is lower than aggregated totals for diagnoses and treatments because total patient count = unique patients and some patients had multiple events within or across sub-groups. A&E = accident and emergency.*

A total of 5122 eye-related and 905 224 general A&E attendances were identified, respectively, resulting in an A&E annual attendance rate of 0.6% for eye-related causes. Presenting reasons are shown in Table 2b.

**Figure 3.**
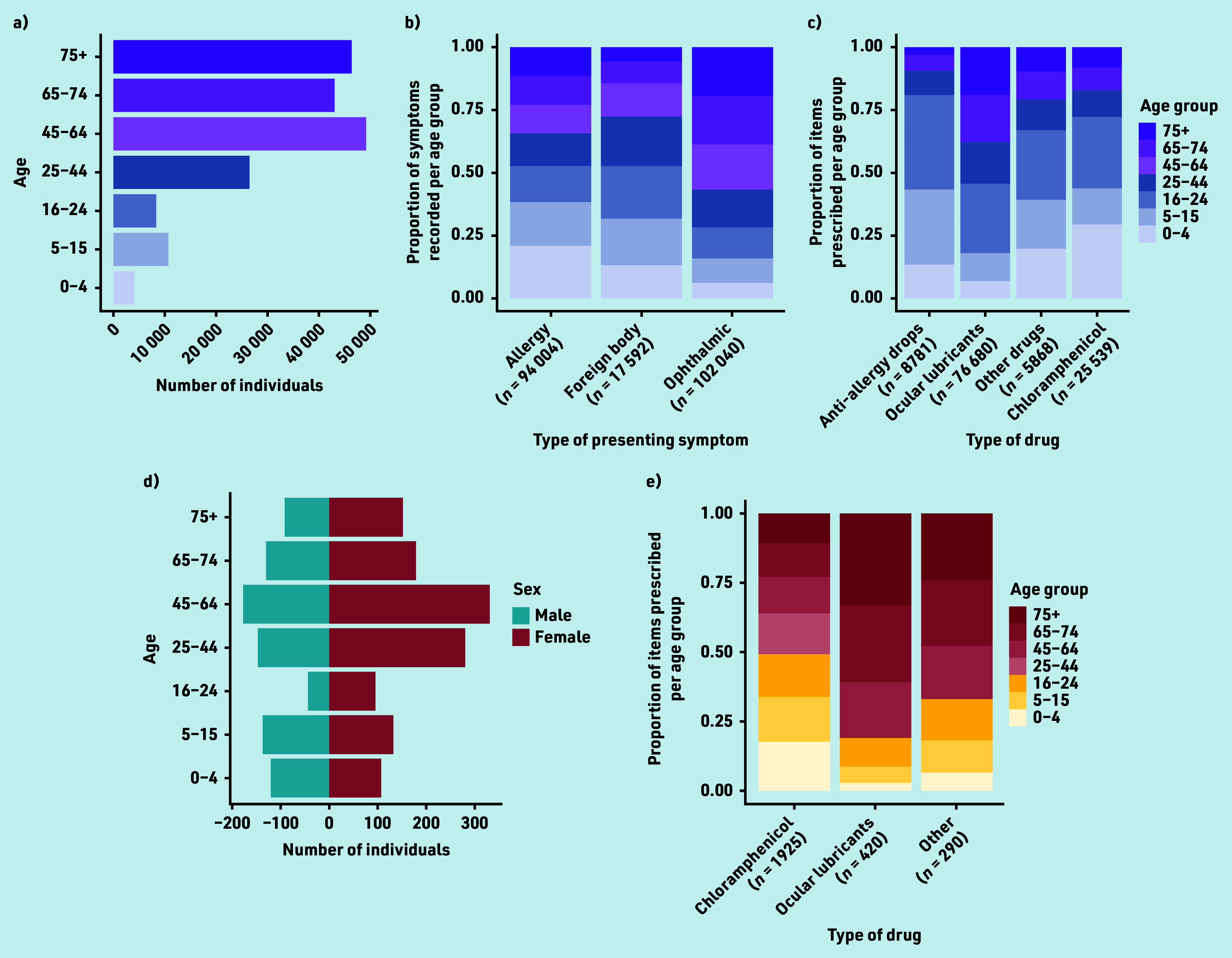
Optometry and pharmacy data. Eye Health Examination Wales (optometry) audit data for the study period showing (a) the number of individuals in each of seven age groups, (b) the proportion of symptoms recorded per age group by symptom type, and (c) the proportion of items issued per age group by drug type. The Common Ailments Scheme (pharmacy) audit data for the study period showing (d) the number of male and female individuals in each of seven age groups and (e) the proportion of items issued per age group by drug type. The presented categorisations of symptoms and type of drug are consistent with those reported in the audit data for the given service, and therefore differ between services.

The 188 960 attendances resulted in 116,868 items being supplied or advised (Figure 3c). Of 2085 attendances to the community pharmacy Common Ailments Scheme, most were females aged >45 years.

All four services collectively managed 365 044 acute eyecare episodes, of which optometrists and pharmacists managed 51.8% (116 868) and 0.6% (2635) of cases, respectively. Services collectively issued 939 926 acute eye medications or prescriptions, of which optometrists and pharmacists issued 12.4% (116 868) and 0.3% (2125), respectively.

### Effects of demographics and socioeconomic status on GP prescribing services

**Figure 4.**
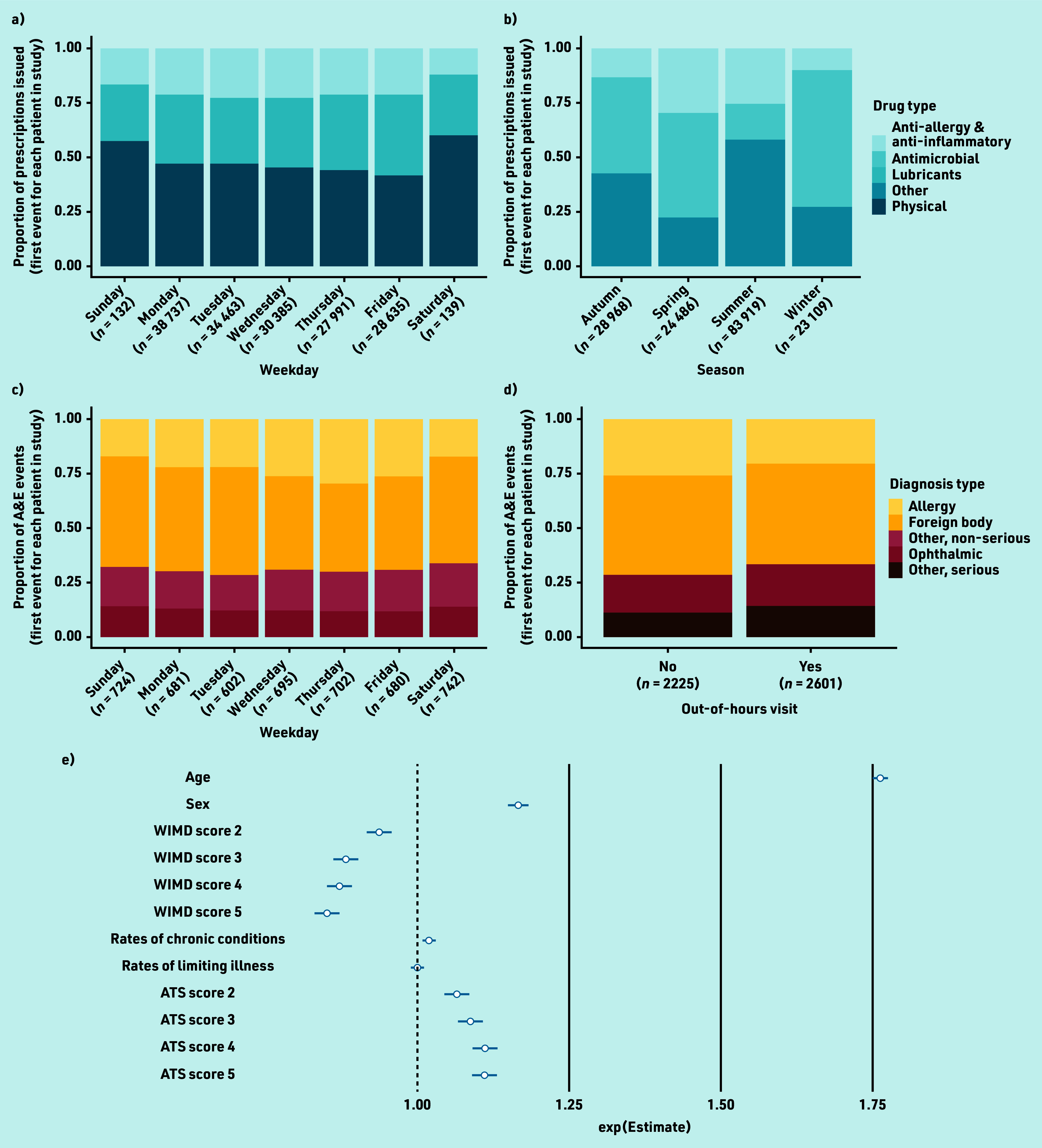
Burden of acute eyecare conditions over time and effect of predictor variables. Burden of acute eyecare conditions on (a) GP prescribing services by weekday classified by drug type, (b) GP prescribing services by season classified by drug type, (c) A&E services by weekday classified by diagnosis type, (d) A&E services by whether the event took place out of hours (outside of 6 a.m.–6 p.m. Monday–Friday) or not. To prevent the disclosure of identifying information, physical and ‘other’ treatments have been removed from (a) and allergy diagnoses have been removed from (c) and (d). Effect sizes of demographic and socioeconomic predictor variables on episode burden of acute eye conditions, measured as the number of prescriptions issued, calculated through quasi-Poisson GLM. Exp(est) = exponentiated Poisson regression coefficient (β) indicating the unit change in the outcome (number of prescriptions) with each unit or level change in each predictor, should all other predictors be held constant (e). Error bars in (e) show the 95% confidence intervals. WIMD levels 2–5 = Welsh Index of Multiple Deprivation quintiles contrasted against the most deprived areas (level 1); chronic conditions = rate of chronic conditions measured within the WIMD; limiting illness = rate of limiting illnesses measured within the WIMD; ATS levels 2–5 = Access to Services domain quintiles contrasted against the most deprived areas (level 1). The presented categorisations of drug and diagnosis type are consistent with those reported in the audit data for the given service, and therefore differ between services. A&E = accident and emergency. ATS = access to services. GLM = generalised linear model.

Quasi-Poisson GLM on total burden revealed significantly positive associations between patient age and episode burden (adjusted Poisson regression coefficient [adjusted β] 1.76, 95% confidence interval [CI] = 1.75 to 1.77, *P*<0.001; Figure 4e; Supplementary Table S1) and between episode burden and female sex (adjusted β 1.17, 95% CI = 1.15 to 1.18, *P*<0.001).

A significantly negative association was observed between episode burden and overall deprivation score, that is, episode burden was lowest in more affluent Lower Layer Super Output Areas (LSOAs; adjusted β 0.94, 95% CI = 0.92 to 0.95, *P*<0.001; Supplementary Table S1). In contrast, a significantly positive association was observed between episode burden and ATS domain scores, that is, episode burden was highest in more highly provisioned connected LSOAs (adjusted β 1.07, 95% CI = 1.05 to 1.09, *P*<0.001; Supplementary Table S1). No association was found between episode burden and rates of chronic conditions or limiting illnesses in a patient’s LSOA.

### Effect of seasonality on likelihood of GP prescribing events

Following data visualisation, it was observed that burden was greatest in summer (52.3%; *n* = 83 919) and lowest in winter (14.4%; *n* = 23 109; Figure 4b). Prescribing of ocular lubricants in summer was particularly high (52.0%; *n* = 48 687). The proportion of anti-allergy and anti-inflammatory drugs issued was greatest during the spring months (29.6%; *n* = 7232). The majority (62.4%; *n* = 14 419) of prescriptions issued in winter were antimicrobials; however, similar counts of antimicrobials were prescribed in summer (*n* = 13 470; 16.1%) and winter.

Logistic regression on total burden by season revealed increased likelihoods of a prescription being issued in spring (adjusted odds ratio [aOR] 1.51, 95% CI = 1.46 to 1.57, *P*<0.001), summer (aOR = 1.63, 95% CI = 1.58 to 1.69, *P*<0.001), and winter (aOR = 1.45, 95% CI = 1.39 to 1.50, *P*<0.001) compared with autumn (*n* = 28 968), despite overall burden being lower in both spring and winter (Supplementary Table S2).

Logistic regression on burden of individual drug types by season revealed that, in summer, the likelihood of anti-allergy (aOR = 7.94, 95% CI = 4.99 to 10.42, *P*<0.001), ocular lubricants (aOR = 5.16, 95% CI = 3.21 to 6.70, *P*<0.001), physical intervention (aOR = 3.44, 95% CI = 2.18 to 5.44, *P*<0.001), and antimicrobial (aOR = 1.48, 95% CI = 0.92 to 1.93, *P* = 0.003) prescriptions being issued were highly significantly elevated over items classed as other. In spring, the likelihood of anti-allergy prescription being issued increased (aOR = 2.01, 95% CI = 1.45 to 3.00, *P*<0.001) and that of ocular lubricants decreased (aOR = 0.56, 95% CI = 0.40 to 0.83, *P* = 0.001) significantly. In winter, the likelihood of antimicrobial prescriptions being issued increased (aOR = 1.59, 95% CI = 1.31 to 2.62, *P* = 0.011) significantly (Supplementary Table S2).

### Effect of day of the week on GP prescribing events

Logistic regression on total burden revealed that prescribing burden was greatest on Mondays (*n* = 38 737; aOR = 1.31, 95% CI = 1.20 to 1.40, *P*<0.001; Figure 4a; Supplementary Table S3) and lowest on Thursdays (*n* = 27 991; reference condition in logistic regression) with a modest increase on Fridays (*n* = 28 635; aOR = 1.09, 95% CI = 1.00 to 1.19, *P* = 0.041). A very small number of prescribing events were issued at weekends (*n* = 271), likely under the out-of-hours GP service for emergency prescribing. During the week, the proportional burden of each prescribing class remained consistent (Figure 4a). At the weekends, ocular lubricants and physical treatments accounted for a greater proportion of prescriptions issued (Figure 4a).

### Effect of day of the week on A&E events

Following data visualisation, it was observed that A&E burden was greater on Saturdays (*n* = 742) and Sundays (*n* = 724) than on weekdays (Figure 4c). Modest increases were observed in burden as a result of ophthalmic conditions and conditions classed as ‘other’ (serious and non-serious) towards the beginning and end of the week, with minima occurring mid-week. The reverse was true for foreign-body trauma.

Logistic regression on total burden revealed that differences in likelihood of any event taking place between days were insignificant (Supplementary Table S4). Logistic regression on burden of individual drug types revealed that foreign-body occurrences were significantly less likely to occur on all days compared with Thursdays. Allergy diagnoses were less likely to occur on Mondays (aOR <0.01, 95% CI = <0.01 to <0.01, *P*<0.001).

### Likelihood of A&E events occurring out of hours

An increase in burden on A&E services was observed and the likelihood of an attendance out of hours increased (*n* = 2601; aOR = 1.30, 95% CI = 1.13 to 1.51, *P*<0.001; Supplementary Table S5) compared with attendances taking place between 6 a.m. and 6 p.m. Monday–Friday (*n* = 2225; Figure 4d). It was also observed that females were more likely to use A&E services out of hours (aOR = 1.36, 95% CI = 1.21 to 1.54, *P*<0.001; Supplementary Table S8). The likelihood of an out-of-hours visit was lower for foreign bodies (aOR = 0.84, 95% CI = 0.76 to 0.93, *P* = 0.001) than for conditions classed as other, non-serious (Supplementary Table S5).

### Effect of patient demographics on likelihood of optometry and pharmacy events

Most items issued by optometrists were ocular lubricants (*n =* 76 680, 65.6%; Figure 3c), mostly to older age groups. Chloramphenicol (*n* = 25 539, 21.9%) and anti-allergy drops (*n* = 8781, 7.5%) were mostly supplied to younger age groups. This age-related trend was also observed for items issued by pharmacies (Figure 3e), who, by contrast, issued chloramphenicol (*n* = 1925) more than ocular lubricants (*n* = 420).

Logistic regressions on individual diagnosis types and drug types revealed statistically significant but negligible effects of age on items issued by optometrists (Supplementary Table S6). Similarly, logistic regression on prescribing by pharmacies revealed a statistically significant but negligible increase in the issuing of ocular lubricants over chloramphenicol with increased age (aOR = 0.98, 95% CI = 0.97 to 0.99, *P* = 0.004; Supplementary Table S7).

## Discussion

### Summary

This national-level population-based study characterised the burden on acute eyecare services across four distinct healthcare services. Optometrists and GPs managed the greatest burden of acute eye cases, with fewer attendances and medicines provision recorded in A&E and pharmacy, respectively. An incidence rate of 65.4 episodes of care per 1000 people per year attending GP and A&E services was found. The prescribing rate for GP services was 0.9%, lower than previously reported UK rates.^4^^,^^9^^,^^33^ This may be explained in part by the well-established Welsh optometrist-led acute eye service, in which patients may attend same-day acute eye appointments via community-based enhanced optometric services. The A&E attendance was 0.6%, identical to that previously reported in the US.^34^

### Strengths and limitations

This study utilised routinely collected administrative data and is limited by completion and linkage accuracy; however, a low level of missing data was observed from the SAIL databank with WIMD data missing for 8833 (5.1%) of individuals. The findings do not represent individuals obtaining medicines from hospitals, without a prescription, from a pharmacy outside the Common Ailments Scheme, or through private medical services. Data on sex were not available in the optometry dataset. It was assumed that each consultation was a separate patient in both optometry and pharmacy datasets. Any dataset using coded data from healthcare professionals may be liable to coding errors and is not subject to code validation in this study.

### Comparison with existing literature

The age and sex distribution in those attending GP and A&E services for acute eyecare represents a unique population relative to that for all conditions. Older females and infants of both sexes were more likely to use GP services, while adolescent and middle-aged males were more likely to visit A&E. This pattern agrees with previous findings in general A&E^15^^,^^35^ and GP services.^36^^,^^37^

A strong relationship between age and episode burden (number of prescriptions issued by GPs and attendances to A&E; adjusted β 1.76, *P*<0.001) was notable. Such relationships may be expected, since the burden on health services is greater among older adults^38^ and dry eye symptoms increase with age.^39^^,^^40^ The older population in Wales is increasing rapidly. An increase of 24% in people aged >75 years is expected over the next decade,^41^ thus the burden reported here is likely to increase in the future.

GPs prescribed anti-allergy and anti-inflammatory drugs more frequently in spring and summer, which is consistent with the symptoms of ocular allergy that typically occur during these months.^42^ The type of prescription did not vary with days of the week for both GP and A&E services, nor did it vary for out-of-hours A&E services compared with daytime hours. This is consistent with reported prescribing of antibacterial eye drops by GPs^43^ and antibiotics by dentists.^44^

Ocular lubricants and antimicrobial drugs accounted for 75.9% (*n* = 623 250) and 9.9% (*n* = 80 921) of all acute eye-related GP prescriptions, respectively. Similarly, optometrists mainly issued ocular lubricants (65.6%; *n* = 76 680) and chloramphenicol (21.9%; *n* = 25 539). Yet pharmacists issued fewer ocular lubricants (15.9%; *n* = 420) relative to chloramphenicol (73.1%; *n* = 1925). This may reflect the availability of ocular lubricants for purchase in the pharmacy without a consultation with the pharmacist, unlike chloramphenicol. Ocular lubricants can be issued for both acute (for example, viral conjunctivitis) and chronic (for example, recurrent corneal erosions) conditions, but are frequently prescribed for dry eye disease, which is a chronic disorder that often presents with acute symptoms.^45^ These commonly prescribed items support literature from the UK^46^ and Canada.^47^ In Wales, 47% of people reported seeking help from their GP for eye pain and redness.^14^ Yet, GP trainees find management of eye conditions challenging^48^ and there is evidence of overprescribing of antibiotics by GPs.^49^ Conversely, optometrists are well placed to manage such conditions in primary care, given their specialist equipment and training.

The study evidences the potential to reduce the burden of acute eyecare services on GPs, given the changing eyecare pathways in the UK. Of the 820 693 prescriptions issued by GPs, 93.3% (*n* = 766 083) were identified as medicines that could have been supplied by entry-level optometrists while 6.7% (*n* = 54 610) of prescriptions would have required IP qualification. Given that optometry and pharmacy services include weekend opening hours, there may be potential to reduce the burden of acute eye attendances to A&E, which is greatest on these days. UK optometry services have responded to increasing eyecare demand through the shift in some eyecare delivery from hospital to community settings^24^ and through upskilling in areas such as therapeutic prescribing,^50^ which can reduce referral to acute hospital services.^51^

Episode burden was found to decrease with increased affluence, consistent with prescribing patterns for antibiotics^52^ and general medicines,^53^ and the greater use of general A&E^35^ and GP^37^ services in more deprived areas. While deprivation is thought to be one determinant of health, it is likely influenced by many complex factors, including social inequalities, for example, income inequalities and differences in health behaviour.^54^^,^^55^

### Implications for research and practice

The findings reflect the well-established link between socioeconomic deprivation and poor health, as well as lower levels of health literacy.^56^^–^^58^ Poor health literacy is associated with poorer outcomes in a range of eye conditions.^59^^–^^61^ Interventions improving health literacy in deprived areas have been successful in other health conditions ^62^^–^^65^ and could similarly contribute to appropriate care-seeking behaviour for acute eyecare. For example, behavioural interventions that are community-based and facilitated by trained peer supporters are aimed at improving awareness of conditions and reducing time to symptom presentation. In the context of acute eye care, this could translate to, for example, awareness campaigns to choose optometry first for a itchy red eye*.* Some patients will continue to present to GP surgeries or A&E departments. While clinical staff report lacking confidence in managing acute eye presentations,^12^^,^^66^ acute ophthalmic triage tools or checklists can facilitate safe and appropriate care.^12^^,^^66^^–^^68^ Examples of such tools include the National Institute for Health and Care Excellence (NICE) ‘Red Eye’ Clinical Knowledge Summary^69^ and the *BMJ* Best Practice Assessment of red eye guidance.^70^ Further exploration of the impact of easier access to ophthalmology specialist advice for community practitioners is warranted. All providers – for example, reception staff and duty doctors in a GP service – should be aware of the capabilities and capacity of other healthcare providers for managing acute eye problems, and practices should review their procedures for supporting patient care navigation to ensure they attend the most appropriate eye care service within the practice or externally at first contact.

This study presents, for the first time to the authors’ knowledge, data combined from four different types of healthcare providers, using national-level population data, to represent the prescribing burden associated with acute eyecare. Acute eyecare continues to place considerable burden on GPs and A&E, particularly in areas with greater economic deprivation and lower overall health. This is likely to increase with a rapidly ageing population. With ongoing pathway development, including weekend optometry and pharmacy services, community-based IP optometrists and pharmacists and patient education, there may be scope to change this trajectory. The evidence highlights the imperative to ensure sufficient workforce planning in optometry and pharmacy to reduce the burden on GPs and A&E. Future evaluations of service improvement efforts must examine for unintended consequences on competence that might be created elsewhere in the system (for example, in general practice).

## Data

This study makes use of anonymised, individual-level data held in the SAIL Databank, a Trusted Research Environment, at Swansea University, Swansea, UK. Due to the nature and level of the data, data are not publicly available. All proposals to use SAIL data are subject to review by the independent Information Governance Review Panel (IGRP). The IGRP gives careful consideration to each project proposal to ensure proper and appropriate use of SAIL data. If a project is approved, access to the requested data is gained through a privacy-protecting safehaven and remote-access system referred to as the SAIL Gateway. SAIL has established an application process to be followed by anyone who would like to access data via SAIL at: https://saildatabank.com/governance/approvals-public-engagement/information-governance/.

## References

[1] Buchan JC, Barnes B, Cassels-Brown A (2017). The urgent need to develop emergency EYE care in the UK: the way forward?. Eye (Lond).

[2] Smith HB, Daniel CS, Verma S (2013). Eye casualty services in London. Eye (Lond).

[3] Siempis T (2014). Urgent eye care in the UK increased demand and challenges for the future. Med Hypothesis Discov Innov Ophthalmol.

[4] Baker H, Ratnarajan G, Harper RA (2016). Effectiveness of UK optometric enhanced eye care services: a realist review of the literature. Ophthalmic Physiol Opt.

[5] Zafar S, Sebestyen K, Qureshi Z (2020). National trends in imaging rates for eye-related emergency department visits in the United States. Am J Ophthalmol.

[6] Lim LW, Yip LW, Tay HW (2020). Sustainable practice of ophthalmology during COVID-19: challenges and solutions. Graefes Arch Clin Exp Ophthalmol.

[7] Wickham L, Hay G, Hamilton R (2020). The impact of COVID policies on acute ophthalmology services — experiences from Moorfields Eye Hospital NHS Foundation Trust. Eye (Lond).

[8] Schneider JE, Scheibling CM, Segall D (2014). Epidemiology and economic burden of conjunctivitis: a managed care perspective. J Manag Care Med.

[9] Sheldrick JH, Wilson AD, Vernon SA, Sheldrick CM (1993). Management of ophthalmic disease in general practice. Br J Gen Pract.

[10] Sheikh A, Hurwitz B, van Schayck CP (2012). Antibiotics versus placebo for acute bacterial conjunctivitis.. Cochrane Database Syst Rev.

[11] Azari AA, Barney NP (2013). Conjunctivitis: a systematic review of diagnosis and treatment. JAMA.

[12] Kilduff C, Lois C (2016). Red eyes and red-flags: improving ophthalmic assessment and referral in primary care. BMJ Qual Improv Rep.

[13] Spafford MM, Jones DA, Christian LW (2023). What the Canadian public (mis)understands about eyes and eye care. Clin Exp Optom.

[14] Welsh Government (2021). Sensory health (eye care and hearing statistics: April 2019 to March 2021).

[15] Channa R, Zafar SN, Canner JK (2016). Epidemiology of eye-related emergency department visits. JAMA Ophthalmol.

[16] Konstantakopoulou E, Edgar DF, Harper RA (2016). Evaluation of a minor eye conditions scheme delivered by community optometrists. BMJ Open.

[17] Rehan SM, Morris DS, Pedlar L (2020). Ophthalmic emergencies presenting to the emergency department at the University Hospital of Wales, Cardiff, UK. Clin Exp Optom.

[18] Fung M, Myers P, Wasala P (2016). A review of 1000 referrals to Walsall’s hospital eye service. J Public Health (Oxf).

[19] Sheen NJL, Fone D, Phillips CJ (2009). Novel optometrist-led all Wales primary eye-care services: evaluation of a prospective case series. Br J Ophthalmol.

[20] Buller AJ (2021). Results of a glaucoma shared care model using the enhanced glaucoma staging system and disc damage likelihood scale with a novel scoring scheme in New Zealand. Clin Ophthalmol.

[21] Ly A, Wong E, Huang J (2020). Glaucoma community care: does ongoing shared care work?. Int J Integr Care.

[22] Hall G, Cork T, White S (2019). Evaluation of a new patient consultation initiative in community pharmacy for ear, nose and throat and eye conditions. BMC Health Serv Res.

[23] Bilkhu PS, Wolffsohn JS, Tang GW, Naroo SA (2014). Management of dry eye in UK pharmacies. Cont Lens Anterior Eye.

[24] Mason T, Jones C, Sutton M (2017). Retrospective economic analysis of the transfer of services from hospitals to the community: an application to an enhanced eye care service. BMJ Open.

[25] Ford DV, Jones KH, Verplancke JP (2009). The SAIL Databank: building a national architecture for e-health research and evaluation. BMC Health Serv Res.

[26] Lyons RA, Jones KH, John G (2009). The SAIL databank: linking multiple health and social care datasets. BMC Med Inform Decis Mak.

[27] Office for National Statistics (2018). Population estimates for the UK, England and Wales, Scotland and Northern Ireland: mid- 2017.

[28] SAIL Databank (2023). Concept Library.

[29] SAIL Databank (2023). PH1237 & PH1238.

[30] Zeileis A, Hothorn T (2002). Diagnostic checking in regression relationships. R News.

[31] Signorell A, Aho K, Alfons A (2023). DescTools: tools for descriptive statistics R package version 09945.

[32] Venables WN, Ripley BD (2002). Modern applied statistics with S.

[33] Mcdonnell PJ (1988). How do general practitioners manage eye disease in the community?. Br J Ophthalmol.

[34] Vaziri K, Schwartz SG, Flynn HW (2016). Eye-related emergency department visits in the United States, 2010. Ophthalmology.

[35] Petersen J, Longley P, Gibin M (2011). Names-based classification of accident and emergency department users. Health Place.

[36] Cumming J, Stillman S, Liang Y (2010). The determinants of GP visits in New Zealand. Aust N Z J Public Health.

[37] Dunlop S, Coyte PC, McIsaac W (2000). Socio-economic status and the utilisation of physicians’ services: results from the Canadian National Population Health Survey. Soc Sci Med.

[38] Atella V, Piano Mortari A, Kopinska J (2019). Trends in age-related disease burden and healthcare utilization. Aging Cell.

[39] Brewitt H, Sistani F (2001). Dry eye disease: the scale of the problem. Surv Ophthalmol.

[40] Vehof J, Kozareva D, Hysi PG, Hammond CJ (2014). Prevalence and risk factors of dry eye disease in a British female cohort. Br J Ophthalmol.

[41] Office for National Statistics (2022). National population projections: 2020-based interim.

[42] Leonardi A, Doan S, Fauquert JL (2017). Diagnostic tools in ocular allergy. Allergy.

[43] Huibers L, Moth G, Christensen MB, Vedsted P (2014). Antibiotic prescribing patterns in out-of-hours primary care: a population-based descriptive study. Scand J Prim Health Care.

[44] Thompson W, McEachan R, Pavitt S (2020). Clinician and patient factors influencing treatment decisions: ethnographic study of antibiotic prescribing and operative procedures in out-of-hours and general dental practices. Antibiotics (Basel).

[45] Bradley JL, Özer Stillman I, Pivneva I (2019). Dry eye disease ranking among common reasons for seeking eye care in a large US claims database. Clin Ophthalmol.

[46] Jonuscheit S, Geue C, Laidlaw R (2021). Towards transforming community eye care: an observational study and time-series analysis of optometrists’ prescribing for eye disorders. Public Health.

[47] Johnson D, El-Defrawy SR, Hollands S (2016). Drug-prescribing patterns among optometrists and nonophthalmologist physicians at a tertiary care centre in Kingston, Ontario. Can J Ophthalmol.

[48] Morgan S, Tapley A, Henderson KM (2016). Australian general practice trainees’ exposure to ophthalmic problems and implications for training: a cross-sectional analysis. J Prim Health Care.

[49] Cherry MD, Tapley A, Quain D (2021). Antibiotic prescribing patterns of general practice registrars for infective conjunctivitis: a cross-sectional analysis. J Prim Health Care.

[50] Spillane D, Courtenay M, Chater A (2021). Factors influencing the prescribing behaviour of independent prescriber optometrists: a qualitative study using the Theoretical Domains Framework. Ophthalmic Physiol Opt.

[51] Cottrell P, North R, Sheen N, Ryan B (2022). Optometry independent prescribing during COVID lockdown in Wales. Ophthalmic Physiol Opt.

[52] Adekanmbi V, Jones H, Farewell D, Francis NA (2020). Antibiotic use and deprivation: an analysis of Welsh primary care antibiotic prescribing data by socioeconomic status. J Antimicrob Chemother.

[53] Frazer JS, Frazer GR (2020). GP prescribing in Northern Ireland by deprivation index: retrospective analysis. Fam Med Community Health.

[54] Jokela M (2015). Does neighbourhood deprivation cause poor health? Within-individual analysis of movers in a prospective cohort study. J Epidemiol Community Health.

[55] Walsh D, Bendel N, Jones R, Hanlon P (2010). It’s not ‘just deprivation’: why do equally deprived UK cities experience different health outcomes?. Public Health.

[56] Marmot M, Allen J, Boyce T Health equity in England: the Marmot Review 10 years on.

[57] Harris J, Springett J, Croot L (2015). Can community-based peer support promote health literacy and reduce inequalities? A realist review. Public Health Research.

[58] Protheroe J, Whittle R, Bartlam B (2017). Health literacy, associated lifestyle and demographic factors in adult population of an English city: a cross-sectional survey. Health Expect.

[59] Schillinger D, Grumbach K, Piette J (2002). Association of health literacy with diabetes outcomes. JAMA.

[60] Muir KW, Christensen L, Bosworth HB (2013). Health literacy and glaucoma. Curr Opin Ophthalmol.

[61] Muir KW, Santiago-Turla C, Stinnett SS (2006). Health literacy and adherence to glaucoma therapy. Am J Ophthalmol.

[62] Moriarty Y, Lau M, Sewell B (2021). Randomised controlled trial and economic evaluation of a targeted cancer awareness intervention for adults living in deprived areas of the UK. Br J Cancer.

[63] Moffat J, Bentley A, Ironmonger L (2015). The impact of national cancer awareness campaigns for bowel and lung cancer symptoms on sociodemographic inequalities in immediate key symptom awareness and GP attendances. Br J Cancer.

[64] Stormacq C, Wosinski J, Boillat E, Van den Broucke S (2020). Effects of health literacy interventions on health-related outcomes in socioeconomically disadvantaged adults living in the community: a systematic review. JBI Evid Synth.

[65] Schaffler J, Leung K, Tremblay S (2018). The effectiveness of self-management interventions for individuals with low health literacy and/or low income: a descriptive systematic review. J Gen Intern Med.

[66] Chuk L-K, Chung JY-M, Lau HH-W (2022). Emergency nurse practitioners’ use of a modified Edinburgh red eye diagnostic algorithm: prospective observational study. Hong Kong J Emerg Med=.

[67] D’Oria F, Bordinone MA, Rizzo T (2020). Validation of a new system for triage of ophthalmic emergencies: the alphabetical triage score for ophthalmology (ATSO). Int Ophthalmol.

[68] Rossi T, Boccassini B, Cedrone C (2008). Testing the reliability of an eye-dedicated triaging system: the RESCUE. Eur J Ophthalmol.

[69] Health and Care Excellence (2021). Red eye. Clinical Knowledge Summary.

[70] Smith J, Severn P, Clarke L (2022). Assessment of red eye. BMJ Best Practice.

